# Chromatomass-Spectrometric Method for the Quantitative Determination of Amino- and Carboxylic Acids in Biological Samples

**DOI:** 10.3390/metabo13010016

**Published:** 2022-12-22

**Authors:** Anna L. Kaysheva, Arthur T. Kopylov, Alexander A. Stepanov, Kristina A. Malsagova, Alexander A. Izotov, Yevgeniya I. Shurubor, Boris F. Krasnikov

**Affiliations:** 1 Biobanking Group, Branch of Institute of Biomedical Chemistry scientific and Education Center, 123098 Moscow, Russia; 2 Centre for Strategic Planning of FMBA of Russia, 119121 Moscow, Russia

**Keywords:** methods of analysis, amino acids, carboxylic acids, high performance liquid chromatography, tandem mass-spectrometry, multiplex methods of quantitative analysis

## Abstract

A highly sensitive method for the qualitative and quantitative determination of amino- and carboxylic acids, as well as a number of urea and methionine cycle metabolites in the studied solutions, is presented. Derivatives (esterification) were obtained for amino acids by their reaction in a solution of 3 N of hydrochloric acid in n-butanol for 15 min at 65 °C and for carboxylic acids by their reaction with phenol in ethyl acetate with 3 N of hydrochloric acid for 20 min at 65 °C. Experimental work on the determination of individual metabolites was carried out using the HPLC-MS/MS method and included the creation of a library of spectra of the analyzed compounds and their quantitative determination. Multiplex methods have been developed for the quantitative analysis of the desired metabolites in a wide range of concentrations of 3–4 orders of magnitude. The approach to the analysis of metabolites was developed based on the method of the dynamic monitoring of multiple reactions of the formation of fragments for a mass analyzer with a triple quadrupole (QQQ). The effective chromatographic separation of endogenous metabolites was carried out within 13 min. The calibration curves of the analyzed compounds were stable throughout the concentration range and had the potential to fit below empirical levels. The developed methods and obtained experimental data are of interest for a wide range of biomedical studies, as well as for monitoring the content of endogenous metabolites in biological samples under various pathological conditions. The sensitivity limit of the methods for amino acids was about 4.8 nM and about 0.5 μM for carboxylic acids. Up to 19 amino- and up to 12 carboxy acids and about 10 related metabolites can be tested in a single sample.

## 1. Introduction

The analysis of amino acids and acetylcarnitines based on tandem mass spectrometry (MS/MS) is one of the most common methods for diagnosing and monitoring the metabolic disorders of amino-, organic-, and fatty acids [1,2]. In research and laboratory practice, MS/MS analysis methods are widely used [3,4]. Using these methods, it is possible to determine the quantitative composition of about 50 diagnostic metabolites in a single biological sample [5]. Based on this amount of data obtained, more than 30 metabolic disorders can be identified [6]. A significant part of these disorders is included in a single diagnostic group that is recommended by the American College of Medical Genetics for ongoing monitoring [6].

Some of these diseases are associated with inborn errors in the metabolism of amino- and organic acids (isovaleric acidemia [MIM 243500], methylmalonic acidemia, propionic acidemia [MIM 606054], type I glutaric acidemia [MIM 231670]), others are associated with lifelong acquired disorders of the metabolic pathways that are responsible for the regulation of amino- and organic acids in qualitative and quantitative compositions (phenylketonuria [MIM 261600], type I tyrosinemia [MIM 276700], alkaptonuria [MIM 203500], 3-methylglutaconic aciduria type I [MIM 250950], maple syrup urine disease [MIM 248600]). For example, abnormal levels of organic acids in the urine can be due to various causes associated with disorders in the urea cycle (e.g., increased levels of orotic acid), fatty acid oxidation (short-chain acyl-CoA dehydrogenase deficiency [MIM 201470], medium-chain acyl-CoA dehydrogenase deficiency [MIM 201450], multiple acyl-CoA dehydrogenase deficiencies [MIM 231680]), energy metabolism (pyruvate dehydrogenase deficiency, fumarase deficiency [MIM 606812], SUCLA2 deficiency [MIM 603921]), purine and pyrimidine metabolism (uridine monophosphate synthetase-1 deficiency [MIM 61389], dihydropyrimidine dehydrogenase deficiency [MIM 274270]), neurotransmission (aromatic L-amino acid decarboxylase deficiency [MIM 608643]), and other etiologies (ethylmalonic encephalopathy [pMIM 602473], Canavan disease [MIM 271900], glutathione synthetase deficiency, glyceroline synthetase deficiency [MIM 266130], type I [MIM 259900] and type II [MIM 260000] primary hyperoxaluria) [7,8,9].

Thus, the clinical indications for the determination of organic acids can be of a different nature and include their monitoring both in the neonatal and at a later age. This is important, for example, for the timely detection of a number of acute diseases (accompanied by hyperammonemia, hypoglycemia, or ketolactacidosis), neurological disorders (such as convulsions, ataxia, hypotension, lethargy, coma, developmental delay, or unexplained mental retardation), as well as a number of other diseases, including, but not limited to unexplained metabolic acidosis, unusual odor, macrocephaly, liver failure, and others [10,11]. Some of these symptoms, including lethargy and acidosis, may, however, be due to exogenous intoxication. In this regard, organic acid analysis can help diagnose conditions caused by ethylene glycol poisoning, ibuprofen overdose, or gamma-hydroxybutyric acid intoxication as opposed to succinic acid or semialdehyde dehydrogenase deficiency [12].

When choosing a method or technique for solving a given problem, usually their characteristics, such as speed, availability, and efficiency, are of decisive importance. The development of a fast and efficient method for the quantitative determination of amino acids in complex biological matrices remains an important scientific and practical task. In clinical diagnostics, various methods for analyzing amino acids are known, but according to most researchers, their main disadvantages are the duration of the analysis (up to 2 h) and high cost [13]. Quite a lot of effort from various scientific and technical groups has been expended on the development of highly efficient and/or high-performance methods for the analysis of amino acids [14,15,16].

In general, amino acid quantitation methods include a range of analytical approaches based on the use of high-performance liquid chromatography (HPLC) in combination with some form of optical detection (UV, fluorescence) and/or MS/MS [17]. For example, one of the most convenient methods for determining amino acids based on HPLC with a fluorescence detector and pre-column derivatization using OPA is the method proposed by Wu and Meininger [18]. Although this method is convenient and highly reproducible, it also has disadvantages. The disadvantages include a rather long analysis time for one sample (about 1 h), a limited number of detectable amino acids, and, in addition, during the operation of the analytical column, the coelution of individual amino acids is periodically observed, which makes it difficult to further quantify them.

For the determination of carboxylic acids based on HPLC-UV, we used an inexpensive, direct, and fast method developed by us earlier [19]. The disadvantages of this method include its high specificity in relation to the analytical column used, as well as the fact that the detection of metabolites is carried out at a wavelength of 210 nm, which, although not often, still requires increased attention when analyzing the results due to the possible coelution of close metabolites.

Based on the foregoing, it seems that MS is perhaps almost the only method that makes it possible to unambiguously identify metabolites of interest. MS usually plays a key role in the analysis of amino acids since it permits not only the distinguishing of analytes by their mass-to-charge ratio (*m/z*) but also uses multiple reaction monitoring (MRM), which significantly increases the selectivity and sensitivity of the analysis. An analysis of the most promising approaches for the quantitative multiplex detection of amino acids in biological samples is presented in Table 1.

In terms of organic acid detection, HPLC/MS provides more accurate and unambiguous identification of individual metabolites than is possible with platforms such as HPLC with a refractive detector (HPLC/refractometer), HPLC/UV, or HPLC with an electrochemical detector (HPLC/ED). Separately, the important role of MS in the identification of a number of metabolites in complex biological matrices, which can potentially contain interfering compounds, can be noted.

Here, several works should be singled out, which describe the main methodological approaches and stages of verification for methods of the analysis of organic acids in biological samples using MS [25,26]. For example, one can note the use of HPLC with reversed phase and UV detection for the analysis of aliphatic and phenolic acids, as well as aldehydes under the conditions of their extraction with methyl tert-butyl ether [27]. Subsequently, this method was modified for the use of UV and MS with a triple quadrupole, which markedly increased the specificity of the analysis [24]. The approach was used to measure aliphatic and phenolic acids, decomposition products of ammonia fibers, as well as their subsequent analysis. Formic and acetic acids were determined using a single quadrupole MS [25].

One of the popular HPLC options is the use of stationary phase chromatography based on a polystyrene-divinylbenzene polymer matrix (BioRad Aminex® HPX-87H, Phenomenex Rezex™-RFQ, Hercules, CA, USA) [28,29]. This type of carrier provides the good separation of simple sugars (glucose, xylose), many organic acids, alcohols (ethanol, n-butanol), as well as sugar degradation products (5-hydroxymethylfurfural, furfural). In this case, only acidified water is used as the mobile phase since the polymer matrix is highly stable over a wide pH range. However, one of the disadvantages of this type of chromatographic column is its high cost. For the analysis of organic acids by MS, the commonly used sulfuric acid is replaced by volatile formic acid or acetic acid [30,31]. Nevertheless, the appearance of some difficulties associated with the possible formation of adduct ionization products and by-products of the esterification reaction cannot be ruled out here.

Usually, organic acids are isolated from biological samples (urine, blood plasma) using organic solvents, and their presence and amount in the sample are determined using GC or HPLC-MS. More than 500 organic acids can be detected in urine [32]. To identify the state of a genetically determined disease of organic aciduria, both qualitative and quantitative methods of analysis are suitable, with careful monitoring and correct evaluation of the data obtained. For example, in classical organic aciduria, diagnostic biomarkers often have elevated concentrations in the sample, so the diagnosis of the disease is possible without quantitative analysis [12]. An analysis of the most promising approaches for the quantitative multiplex detection of carboxylic acids in biological samples is presented in Table 2.

It is important to note that in some diseases, biomarker levels may be close to the normal values observed in clinically healthy patients [39,40]. Therefore, it is imperative to conduct a thorough study of the chromatograms for the presence of clinically significant compounds, including those that are present in small quantities or are hidden due to coelution with other compounds.

The use of a program that monitors compliance with laboratory requirements for the analysis of organic compounds in clinical diagnostics contributes to the timely detection of undiagnosed diseases. Often this is due to the difficulty of detecting compounds that are important for the detection of pathognomonic symptoms but are present in the sample at low concentrations [41]. The quantitative analysis of the main biomarkers of a particular disease can also be useful for the therapeutic monitoring of previously diagnosed patients [32,42,43,44,45].

However, unfortunately, modern methods for the quantitative determination of amino acids (Table 1) and carboxylic acids (Table 2) are mostly based on the use of expensive isotope-labeled target metabolite standards and/or expensive chromatographic columns for isolating target metabolites, which, according to a number of specialists, is one of the limiting factors for the development and wider implementation of this methodology in practice [1,2,3,4].

The present work is devoted to the creation of a fast multiplex (HPLC-MS/MS) method for efficient chromatographic separation and the subsequent detection of amino- and carboxylic acids without the use of expensive isotopically labeled standards and costly chromatographic columns for the preliminary isolation of metabolites. The novelty of our work lies in the creation of multiplex methods that are characterized by the high sensitivity and accuracy of analysis while maintaining high speed, which provides high performance at very moderate costs for analysis.

The calibration curves obtained for the analyzed compounds are characterized by high stability over the entire range of concentrations and can potentially be approximated in the region of concentrations below the empirical ones. Analytical indicators, such as the detection limit, of the methods proposed in the work are improved compared to similar studies while maintaining the speed of the method (13.5 and 12 min for amino acids and carboxylic acids, respectively).

## 2. Materials and Methods

### 2.1. Reagents

Water, HPLC grade (ITW Reagents PanReac®, cat. no. 361074.1612, AppliChem GmbH, Darmstadt, Germany), acetonitrile, HPLC grade (Carlo Erba Reagents S.A.S, cat. no. 412329000, Emmendingen, Germany), formic acid (Sigma-Aldrich, cat. no. 33015, St. Louis, MO, USA), trifluoroacetic acid (Acros Organics, cat. no. P39720010, Thermo Fisher Scientific, Waltham, MA, USA), heptafluorobutyric acid (Acros Organics, cat. no. 172801000, Thermo Fisher Scientific, Waltham, MA, USA), ammonium acetate (Fluka, cat. no. 73432, Buchs, Switzerland), methanol (J.T. Baker, cat. no. 8402, Radnor, PA, USA), amino and keto acid standards (Sigma Aldrich, cat. no. A9906, St. Louis, MO, USA), human coagulation factor VIII (Sigma Aldrich, cat. no. H0920000-3EA, St. Louis, MO, USA).

### 2.2. Analytical Equipment

For the analysis of the metabolites, we used a high-resolution quadrupole TOF MS (Agilent, Paolo Alto, CA, USA, model G6550 iFunnel, 2013) and a low-resolution triple quadrupole MS (Agilent, Paolo Alto, CA, USA, model G6490A, 2014).

### 2.3. Standards

The list of standards with main identifiers is presented in Supplementary Materials, Table S1.

### 2.4. Esterification of Amino Acids

To carry out the esterification process, the arbitrary weights of each amino acid were taken into separate test tubes. Molecular weight, mass, and final concentrations of amino acids are presented in the Supplementary Materials, Table S2. A fixed volume (0.5 mL) of a 0.1 M HCl solution was added to the prepared amino acid samples. Then, in the resulting solution, the final concentration was calculated for each amino acid.

An equimolar mixture of amino acids with a final partial molar concentration of each amino acid equal to 1 mmol/L was prepared in a final volume of 1 mL of the surrogate matrix (1% solution of human coagulation factor VIII). To do this, the corresponding volumes of each amino acid solution were mixed by bringing the resulting volume with a 0.1 M HCl solution to 1 mL.

After the addition of all the amino acids, the final volume of the equimolar mixture was 845.41 µL. The indicated volume was adjusted to 1 mL by adding 154.59 of a 0.1 M HCl solution. The resultant solution contained an equimolar concentration of each amino acid.

For the effective MC detection of metabolites, free amino acids were derivatized (esterified) in accordance with the recommendations [46,47] with a solution of 3M HCl in n-butanol. For this, 500 μL of an equimolar solution of free amino acids was taken and dried under a vacuum at a temperature of 30 °C for 60 min. The dry residue of the mixture was reconstituted in 300 μL of a solution of 3 M HCl in n-butanol. A solution (1 mL) of 3 M HCl in n-butanol was prepared from 11 M HCl by mixing 257.70 µL of 11 M HCl and 742.30 µL of n-butanol (based on the density of the 11 M HCl solution equal to ρ = 1.179 g/cm^3^). The reaction mixture was incubated at 65 °C for 15 min with constant stirring at 1200 rpm. The excess n-butanol from the reaction mixture was removed by evaporation under a vacuum at a temperature of 30 °C for 40 min in V-AL mode. The resultant dry residue of esterified amino acids was reduced in 0.5 mL of a solution of 0.1% formic acid, 20% acetonitrile, and 0.02% heptaphorbutyric acid to the calculated concentrations of each esterified amino acid in the working solution of 1 mM/L.

### 2.5. Esterification of Carboxylic Acids

To analyze organic carboxylic acids, they were initially transformed into phenolate derivatives. This made it possible to carry out MS detection under the conditions of chromatographic separation on a C18 column of the stationary phase and with positive ionization. To carry out the process of esterification of carboxylic acids, an arbitrary sample of each acid was weighed and dissolved in a fixed volume (0.5 mL) of an aqueous solution of 0.05% formic acid (Supplementary Materials, Table S3). To compile an equimolar mixture of carboxylic acids in a volume of 1 mL, the molar concentration of the resulting solution and the required volume was calculated.

The resulting volumes of carboxylic acids were combined. The total volume of the mixture of carboxylic acids was 398 µL of the surrogate matrix (1% solution of human coagulation factor VIII). To obtain 1 mL of an equimolar solution of acids, 602 μL of an aqueous solution of 0.05% formic acid was added. The derivatives were obtained by reacting phenol in ethyl acetate with 3 M HCl at 65 °C for 20 min. The organic phase was then dried under a vacuum and resuspended in 0.1 M HCl; the total volume was adjusted to 1 mL.

### 2.6. Biological Samples

The study used biological samples of the primary human cell culture of normal lung epithelial cells (HLF) at 6 cell growth time points: 0 h, 24 h, 48 h, 72 h, 1, and 2 weeks. Cells were disrupted by sonication in two volumes of water. Thereafter, five volumes of methanol were added. The resulting suspension was evaporated under 30 °C, the dried pellet was resuspended in 0.1 M HCl, and the total volume was adjusted to 1 mL. The cell lines for the study described in the paper were provided by the National Medical Research Center of Oncology of the Ministry of Health of Russia. The work on the creation of primary cell lines from the tissues of patients was approved by the Local Ethics Committee of the National Medical Research Center of Oncology of the Ministry of Health of Russia, Protocol No. 6/1, dated 10 February 2020. All patients signed informed consent.

### 2.7. Conditions for Chromatographic Separation and Triple Quadrupole Mass Spectrometric Signal Detection

During the entire analysis, the samples were stored in an automated injector sampler at a temperature of 4 °C. The sampling rate was 80 μL/min, and the injection rate was 120 μL/min. The limit of the maximum operating pressure of the pump was 600 bar, flow rates, 0.3 mL/min, and flow acceleration with a gradient up to 100 μL/min. The mobile phases used for the chromatographic separation of the metabolites were as follows: mobile phase (A), 0.1% formic acid, 0.02% heptafluorobutyric acid aqueous solution; mobile phase (B), 0.1% formic acid, 0.02% heptafluorobutyric acid, 90% acetonitrile, 10% methanol. The method for the elution of amino acids from the stationary phase is presented in Table 3.

The total analysis time, including the time to equilibrate the chromatographic column, was 13.5 min. During the analysis, a flow rate was applied at 0.3 mL/min. During periodic washings of the chromatographic column, a gradient method was used to feed the mobile phase with a high content of acetonitrile and methanol (phase B). This approach made it possible not only to increase the washing rate of the chromatographic column but also to partially compensate for the consumption of the washing liquid.

When developing a method for detecting amino- and carboxylic acids in a biological matrix, a Jet Stream was used as an ionization source. The measurements were carried out under positive ionization conditions. The flow rate of the drying gas (nitrogen) was 14 L/min at 300 °C. The flow rate of the focusing gas (nitrogen) was 11 L/min at 280 °C. The voltage on the capillary was 3600 V, and the focusing voltage was 750 V. The potential of the fragmentor corresponded to 380 V, and the amplitude of the potential on the octapole was 750 V × (p/p). The threshold noise level for the frontal scan was 40 u (N), and the threshold noise level for the tandem scan was 45 u. The scanning range of the mass-charge quantities varied from 70 to 700 *m/z*. The isolation of protonated ions was carried out using a quadrupole mass filter with an isolation window width of 0.7 Th (±0.35 Th). The scanning speed in frontal mode was 2.14 spectra per second, the scanning speed in tandem mode was 4.97 spectra per second, and the total time of one complete scan cycle was 250 s.

### 2.8. Conditions for Chromatographic Separation and Quadrupole Time-of-Flight Mass Spectrometric Signal Detection

The analysis of the total metabolome fraction was conducted on a high-resolution quadrupole time-of-flight mass spectrometer Xevo G2-XS (Waters, Elstree, UK) equipped with a Z-spray electrostatic ionization source and operated in a positive ionization mode at a 2.8 kV capillary voltage and 17 V cone voltage offset to 23 V. The desolvation gas flow rate was adjusted to 600 L/h at a temperature of 375 °C, and the cone gas flow was 50 L/h at a temperature of 150 °C. A precursor ions survey was conducted in a sensitivity analyzer mode within the range of 50–700 *m*/*z* within 140 ms of the full duty cycle. Fragment ions were obtained after decomposition in the CID mode at collision energy ramping within 20–47 eV, using argon as a collision gas. The acquisition was supported with an active online calibration by the lock-mass at *m*/*z* = 556.27 (Leu-Enkephalin at 50 pg/µL) and at *m*/*z* = 309.11 (Warfarin at 250 pg/µL), which were both injected at a flow rate of 5 µL/min and acquired every 30 s with an isolation tolerance of 3 mDa.

The samples were separated using an Acquity H-Class UPLC system (Waters, Elstree, UK) and loaded in a volume of 5 µL onto an Acquity™ UPLC BEH C18 (2.1 × 50 mm, 1.7 µm particle size; Waters, Elstree, UK) column with the pre-installed in-line 0.2 µm filter at a flow rate of 0.3 mL/min. The column was heated at 50 °C. Samples were separated in a gradient of mobile phase A (water) and mobile phase B (acetonitrile), both supplied with 0.1% formic acid using the following gradient scheme: 0–2.5 min 3% of B, then raising the B to 17% at 4 min, before, raising the B to 60% at 5.7 min and rapid increasing of B to 97% at 6 min. The washing B was kept in the isocratic mode until 7.5 min at 0.45 mL/min flow rate, following a smooth decrease to the initial condition at 8.2 min and equilibration for the next 1.3 min at 0.3 mL/min.

## 3. Results

### 3.1. HPLC-MS/MS Measurements of Amino Acids and Their Derivatized Forms

**Figure 1:**
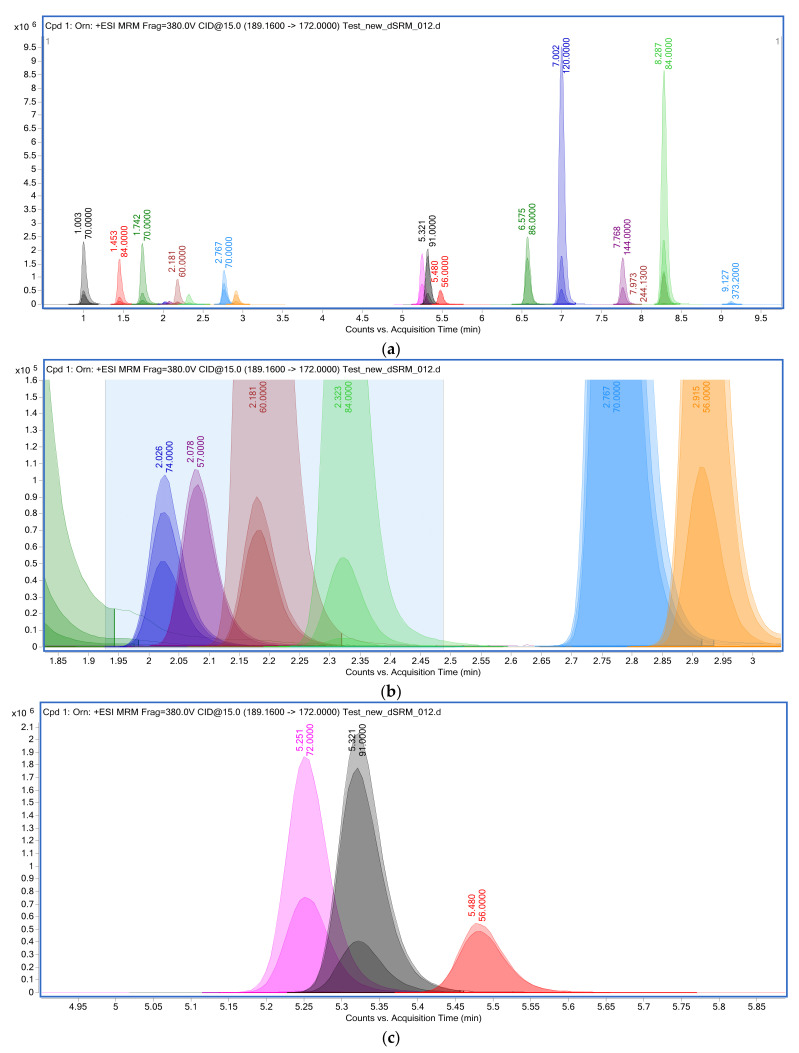
Chromatogram of esterified amino acids by isolated XIC/EIC ion current under detection conditions by monitoring multiple reactions of the formation of SRM fragment ions. Top panel (**a**) shows a general view of the chromatogram in the metabolite separation interval from 0 to 9.5 min. Middle panel (**b**) shows an enlarged gradient region from 1.9 to 2.5 min, corresponding to column retention times of asparagine RT = 2.026 min, glycine RT = 2.078 min, serine RT = 2.181 min, and glutamine RT = 2.323 min. The lower panel (**c**) shows an enlarged section of the gradient interval from 4.95 to 5.8 min, corresponding to the retention time on the column of valine RT = 5.251 min, tyrosine RT = 5.321 min, and methionine RT = 5.480 min.

In the developed procedure for determining amino acids on the HPLC-MS/MS platform, the esterified forms of metabolites were detected as monoisotopic ions with the ionic formula [M+H]^+^. The time of one complete scanning cycle was 0.325 s at a rate of 6.153 scans per second and took place in the frontal (MS1) scanning mode of mass-charge quantities in the range *m*/*z* 50–600 (Figure 1). The main chromatographic and MS characteristics of the protonated ions of esterified amino acids are presented in Table 4. Monoisotopic masses of amino acids are expressed in units of *m*/*z* and are corrected for the alkoxy group.

The limit of inter-experimental accuracy for the retention time in repeated assays (n = 5) ranged from 0.08% (minimum value, tyrosine) to 0.93% (maximum value, asparagine), which corresponds to the standards of retention time accuracy within ±1%. The linear range of the chromatographic peak areas in the equimolar mixture of esterified amino acids was 1,328,245 charges × sec. The average value of the full width of the chromatographic peak at half the maximum height was 5.82 ± 1.34 s.

The stability of the mixture of derivatized amino acids was evaluated. A solution with an equimolar concentration of 3 µmol/L contained in a temperature-controlled chamber of an autosampler at a constant temperature of 6 °C was tested. Peak areas were determined in five repetitions at 24, 72, and 96 h, respectively. A comparison of the obtained data was carried out with respect to the values of the areas of the peaks obtained in the analysis of the conditionally initial reference point of sample storage (T = 0) (Table 5).

According to the presented data, most of the amino acids, throughout the entire storage time, were highly stable. The maximum degradation was observed for methionine—34%, alanine—24%, tryptophan, and threonine—both by 17%. At the same time, in the case of alanine, losses of 13% were observed already after 24 h of storage. In the case of methionine and tryptophan, storage losses were most likely due to amino acid oxidation over time.

Table 6 shows the retention time on the column of the detected amino acids with a tolerance window of ± 0.3 min (18 s).

Despite the close retention time of some amino acids on the column, which differed by less than 0.1 min, almost all of them were successfully resolved. Thus, due to the resolution of the ion current in Table 6, the data obtained can be used for the quantitative analysis and construction of calibration curves.

Table 7 presents the characteristics of the calibration curves for the individual amino acids. Calibration curves were plotted by the equal current linear correlation using a weighting factor of 1/x or 1/x^2^. Moreover, for some amino acids, all seven concentration levels of calibration by 21 points were used. Calibration curves were constructed as a correlation between the relative signal response of the quantifier peak area of the measured amino acid and the expected amino acid concentration relative to the internal standard under the conditions of the presented calibration level.

An exception was made only for glutamic acid, where the calibration curve was built using five calibration levels and 15 points. The two upper concentration levels of 5 and 2.5 µmol/L were excluded from consideration since they reached the logarithmic saturation phase of the calibration curve and, thus, were the upper detection limit for the considered amino acid in Table 7.

The obtained calibration curves permit the quantitative analysis of amino acids to concentration levels of 0.4–1.0 ng/mL. At the same time, the lower limit of detection was not reached by any of the analyzed amino acids. The sensitivity of the quantitative method used made it possible to approximate the results at least in an order of magnitude below the achieved concentration limit from the calibration curve.

### 3.2. HPLC-MS/MS Measurements of Carboxylic Acids and Their Derivatized Forms

Table 8 presents the main chromatographic characteristics of the detected carboxylic acid derivatives with specific values of retention time on the column, relative retention time normalized to lactic acid, asymmetry factor, and peak width at half maximum height expressed in seconds. The *m*/*z* values that were empirically recorded for the analyzed carboxylic acids are also indicated. For citrate and isocitrate, *m*/*z* is indicated, corresponding to the substitution of one and three bases, respectively.

It follows from the data presented in Table 8 that all carboxylic acids, including isomers, were successfully separated by chromatography. The average value of the width of the chromatographic peak at half its maximum height was ω0.5 = 3.35 ± 0.49 s, which fully corresponds to and even exceeds the chromatographic resolution of the column, which was determined earlier when testing on a standard sample.

### 3.3. Development of Multi-Parameter Methods for Quantitative HPLC-MS/MS Monitoring of Multiple Reactions Analysis of Carboxylic Acids in Solution

**Figure 2:**
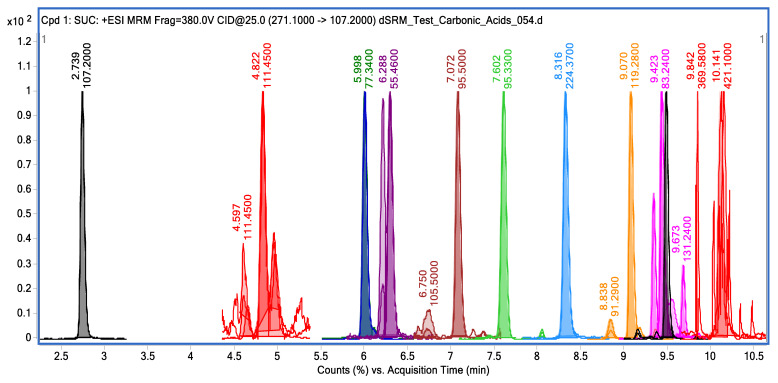
Chromatographic separation of derivatives of carboxylic acids. The specific retention times for each of the carboxylic acid derivatives on the column are shown in Table 8. The peak of lactic acid, in relation to which the retention time on the column was normalized, is indicated in the figure in red, with a retention time of 4.822 min.

For each of the carboxylic acids, the spectra of monoisotopic mass, decomposition by primary fragment ions with the ranking of activation energy, and adducts of target metabolites were obtained, if applicable to the conditions of the chromatographic separation. Figure 2 shows the chromatogram of carboxylic acid derivatives.

Table 9 shows the main characteristics of the calibration curves for each of the considered carboxylic acids. All calibration curves were fitted to a linear regression function within four orders of magnitude. The correlation coefficient for all the carboxylic acids presented varied within the linear range of the calibration curve from r^2^ = 0.9554, which was the minimum value for taurine over seven calibration levels, to r^2^ = 0.99 for the vast majority of the analyzed carboxylic acids.

Calibration curves are plotted over four orders of magnitude spanning seven calibration levels. Technical repetitions within the same calibration level were averaged if the standard deviation in the peak area of the quantifier ion did not exceed ± 10%. For succinic, lactic, malic acids, and taurine, calibration curves were drawn over six concentration points, excluding the upper end of the calibration curve.

From the data indicated in Table 9, it follows that there was no need to use a weight factor to construct the calibration curves for the studied carboxylic acids. The exceptions were isocitric acid and taurine, where for a more satisfactory correlation, a weighting factor 1/x was used; the correlation coefficients without using the factor 1/x were r^2^ = 0.9137 for isocitric acid and r^2^ = 0.8879 for taurine.

### 3.4. HPLC-MS Analysis of Metabolites in Cell Culture Samples

To assess the precision of the developed method when measuring the metabolites in a biological matrix, we conducted an analysis of the primary HLF and calculated the coefficients of variability (CV, %) for each metabolite in each cell sample (Table 10). A total of 42 metabolites were identified in HLF at six cell growth time points: 0 h, 24 h, 48 h, 72 h, 1, and 2 weeks (252 values). The measurements for each of the metabolites present in the table were carried out in two technical repetitions. The calculation of the CV values for two technical repetitions was carried out as described previously [48].

The CV of individual metabolites in the measurements ranged from 0 to 9.3%. The mean CV variability of all the measurements for six-cell growth points ranged from 2.7 to 3.4%. At the same time, the proportion of metabolites with a variability of 0–5% was 70–90%, and the proportion of metabolites with a variability of 5–10% was a smaller part (10–30%). It should be noted that the data on technical repetitions demonstrate an excellent result in terms of the accuracy of the presented analysis, where most of the measurements (up to 90%) had a variability below 5%. According to Gegner et al., CV ranges from 0 to 10% are considered excellent, 11–20% are considered good, 21–30% are considered acceptable, and >30% are not acceptable [49]. Other authors, regarding data on the accuracy of measurements of amino acids in biological samples, mentioned that the precision of similar measurements was in the range of 15% [50].

Thus, our results indicate a fairly high accuracy of the method developed by us and correspond to the concentration range of the same metabolites measured by the previously developed methods.

## 4. Discussion

The analysis of the content of free amino acids is associated with a number of difficulties. Due to the zwitterionic properties of amino acids and the rather wide range of the relative hydrophobicity of metabolites, it is extremely difficult to separate them using standard reverse phase chromatography. In many cases, it is necessary to use ion pair chromatography, the derivatization of free amino acids, esterification with butyl alcohol, or a combination of these two approaches [23,51]. The ion-pair chromatography of free non-derivatized amino acids or the reverse-phase chromatography of esterified amino acids does not provide a satisfactory separation or retention of polar amino acids in the stationary phase. In addition, such chromatography does not sufficiently separate amino acids from other organic compounds in the biological sample, resulting in the significant ion suppression of the signal. The esterification of carboxyl groups facilitates the process of protonation since, in this case, only the amino group remains, which is capable of ionization at given activation energies [51]. At the same time, chromatographic separation becomes more efficient due to a decrease in the hydrophilicity of the analyte due to the presence of a butyl ester group and the interaction of a negative ion-pair component in the mobile chromatographic phase with a positive charged free amino group of amino acids. In the case of keto acids or diketones, derivatization is carried out along the path of forming Schiff bases or hydrazone derivatives, after which esterification is carried out, in particular, with butanol in an acid medium.

This article presents the results of a quantitative analysis of derivatized forms of amino and carboxylic acids using chromato-mass spectrometry. We have adapted derivatization methods and created HPLC–MS/MS libraries of spectra for the compounds of amino acids and carboxylic acids on Q-TOF time-of-flight quadrupole MS, which are now available to interested researchers. Through the method of the dynamic monitoring of multiple reactions of the formation of dSRM fragments, a quantitative analysis of metabolites of interest in the range of 3–4 orders of concentration was carried out on a mass analyzer with a triple quadrupole QQQ. The main comparative characteristics of the methods for the detection of amino and carboxylic acids are shown in Table 11.

The permissible deviation of the concentration measurement accuracy for all the concentration points did not exceed σ = ±10% (limits from 90% to 110% in terms of the reproducibility of the chromatographic peak area, as a value reflecting the concentration of the measured analysis).

Analytical values were calculated according to the Protocols for the Determination of Limits of detection and Limit of Quantitation, Approved Guideline. CLSI document EP17. Wayne, PA USA: CLSI; 2004 [52].

Thus, lowering the signal detection limits and approximating the calibration curves below the achieved limits is inappropriate since the achieved sensitivity of the detection and quantification method is significantly lower than the actual physiological concentrations of free amino acids in biological samples. This feature of the method may be in demand, for example, for the analysis of biological samples in cases of critical limitation in the amount of tissue, for example, a biopsy. On the other hand, when analyzing foodstuffs, drinks, etc., it is desirable to identify compounds that may potentially have the ability to accumulate in organs and tissues. For example, ketoglutaramate in the lactam form is not metabolized and, for the most part, is not excreted from the body. The accumulation of this metabolite is observed in some cases of neuropathology and other diseases [53,54].

For both the carboxylic acids and amino acids considered in this work, the lowest detection limit was not reached. At the same time, the expediency of such an approach is not obvious since the physiological levels of the analyzed carboxylic acids are within the approximated calibration curves. To refine the quantitative data, it would be reasonable to approximate the concentrations of the calibration points within the presented range, with a dilution of 1:2.

For the analyzed acids, the value of the signal-to-noise ratio SNR at the lower level of the calibration curve significantly exceeded the SNR > 5 limits set in the “Signal Identification Criteria”. The minimum signal-to-noise level was recorded for lactate. At the bottom of the calibration curve, corresponding to 0.046 mg/L (0.5 μM) lactate, SNR = 279.84 is the average of the three technical repetitions, calculated as the root mean square within ±0.3 min from the apex of the chromatographic peak.

A significant excess of the actual level of signal-to-noise over the maximum allowable values at the lower points of the calibration curve indirectly testify in favor of the calibration approximation potential by 2–3 orders of a magnitude lower than the achieved signal detection limits.

## 5. Limitations

The proposed method may require the additional purification of the sample during its pre-treatment before analysis in order to minimize or completely eliminate the possible interference or cross-reactivity with compounds possessing similar properties. On the one hand, similar to other label-free methods, this approach is slightly less accurate than methods that use internal standards with stable isotopes, but the calculated concentrations of amino acids and carboxylic acids fall within the expected concentration range. On the other hand, incomplete derivatization, or incomplete recovery after derivatization, especially for carboxylic acids, can lead to a possible overrepresentation of isobaric compounds. Thus, these reactions require optimization and control to maintain the specificity and the formation of by-products.

## 6. Conclusions

This study presents HPLC-MS/MS methods for the qualitative and quantitative analysis of the levels of endogenous metabolites, amino acids, and carboxylic acids, as well as individual metabolites of the urea and methionine cycles. The presented method of analysis, which is characterized by high accuracy, was developed to determine the physiological levels of important metabolites in the studied solutions. Together with QQQ multiparametric detection methods, libraries for the spectra of analyzed compounds obtained on a Q-TOF time-of-flight quadrupole mass spectrometer are presented. The method of quantitative analysis includes the concentration of the analyzed metabolites in the range of 3-4 orders. The effective chromatographic resolution of endogenous metabolites occurs in a short, ~13 min interval. The calibration curves of the analyzed compounds are highly stable over the entire concentration range and have the potential to be approximated to concentration ranges below the empirical ones.

## Figures and Tables

**Table 1:** Comparative analysis of HPCL-MS/MS approaches for quantitative detection of amino acids in biological samples.

No.	Number of Compounds	Type of Sample *	Method	Analysis, Min	Method Features	LLOD (μM)	LLOQ (μM)	Ref.
1	33	Plasma	LC-QQQ **	15	–	0.01-2.5	0.01–10	[20]
2	20	Protein components of plant	LC-QLIT ***	8	^13^C/^15^N-labeled standard	0.1–11	0.12–35	[21]
3	28	Serum, plasma, urine, cerebral spinal fluid, and dried blood spot	2D-LC-MS/MS	15	PGC-LC ****	≤0.1	–	[20]
4	20	Mouse plasma	LC-QQQ	13	^13^C/^15^N-labeled standard	0.1–3	–	[22]
5	26	Plasma, urine, cerebral spinal fluid	LC-QQQ	11	ATRAQ *****	–	2.5–10	[23]
6	6	Serum	LC-QQQ	11	–	0.001–0.0025	0.003–0.007	[24]

Type of sample *—what type of biomaterial is the approach intended for; QQQ **—triple quad mass spectrometer; QLIT ***—quadrupole-linear ion trap; PGC-LC ****—porous graphitized carbon-liquid chromatography; ATRAQ *****—kit for amino acid analysis of physiological fluids with amine-reactive isotope-coded tags.

**Table 2:** Comparative analysis of HPCL-MS/MS approaches for quantitative detection of carboxylic acids in blood samples.

No.	Number of Compounds	Type of Sample *	Method	Analysis, Min	Method Features	LLOD (μM)	LLOQ (μM)	Ref.
1	3	Mouse tissue	LC-QTOF **	6	^13^C-labeled standard	0.005	–	[33]
2	30	Cell culture media, cell extracts, tissue extracts, blood, urine	LC-QQQ ***	21	HILIC ****	–	<5	[34]
3	59	Human melanoma cells	LC-QQQ	20	^13^C/^15^N-labeled standard	–	–	[35]
4	7	Human red blood cell	LC-Orbitrap	>10	^13^C-labeled standard	–	–	[36]
5	10 (total 138 metabolites)	Yeast	LC-QQQ	36	–	0.001–3.7	–	[37]
6	10	Rat plasma	LC-QQQ	21.5	–	0.01–0.25	0.01–0.1	[38]

Type of sample *—what type of biomaterial is the approach intended for; QTOF **—quadrupole time-of-flight; QQQ ***—triple quad mass spectrometer; HILIC ****—hydrophilic interaction chromatography.

**Table 3:** Scheme of the elution of amino acids from a chromatographic column Zorbax RRHD Eclipse Plus C18.

Time, Min	% A	% B	Flow Rate, µL/Min
00.0	98	2	0.30
01.7	98	2	0.30
02.2	85	12	0.30
06.0	73	27	0.30
07.8	55	45	0.30
09.0	55	45	0.30
11.0	2	98	0.30
12.5	2	98	0.30
12.7	30	70	0.30
13.0	98	2	0.30
13.5	98	2	0.30

**Table 4:** Chromatographic characteristics of derivatized amino acids obtained using time-of-flight quadrupole MS.

AA	Chromatographic Characteristics *				*m*/*z*
	RT, Min	FWHM, s	As	A, *cnts × s*	
Orn	1.003	4.61	1.13	666771	222
Ala	1.091	3.12	1.08	189398	146
Lys	1.548	4.12	1.06	1195030	146
Arg	1.846	6.15	0.87	1109451	260
Gly	2.128	7.39	1.89	1515129	212
Asn	2.138	5.47	0.91	286435	162
Ser	2.299	6.39	0.75	832514	174
Gln	2.468	3.12	1.08	1054774	132
Cit	2.747	7.19	0.83	894130	188
Thr	3.027	6.24	1.19	1604746	206
Val	5.416	8.73	0.91	1391872	246
Tyr	5.521	5.91	1.09	972054	203
Met	5.637	6.39	1.23	366957	231
Ile	6.499	6.38	0.91	7682444	188
Leu	6.611	8.06	0.84	276501	176
Phe	6.996	5.15	1.01	1180098	172
Asp	7.884	4.89	1.21	1168386	189
Trp	8.278	4.22	0.88	410052	238
Glu	8.352	5.69	1.69	695427	203

* Amino acids are presented in ascending order of retention time (RT). The value of the mass charge state (*m/z*) is given for a monoprotonated ion (all amino acids). The peak width is given to half the maximum height of the chromatographic peak (FWHM). The area is equal to the product of the peak width (As) and the number of elementary unit charges per sec (A).

**Table 5:** Degradation of derivatized amino acids in solution when stored at 6 °C for 24, 72, and 96 h. Data obtained using time-of-flight quadrupole MS *.

AA	T = 24 h	T = 72 h	T = 96 h	Degradation, %
Ala	0.87	0.81	0.76	24
Lys	1.02	1	0.99	1
Arg	1.06	0.97	0.94	6
Asn	1	0.94	0.91	9
Gly	1	0.98	0.95	5
Ser	1.03	0.99	0.98	2
Gln	0.97	0.96	0.93	7
Thr	0.94	0.89	0.83	17
Val	0.99	0.98	0.95	5
Tyr	0.95	0.92	0.9	10
Met	0.9	0.74	0.66	34
Leu	1.01	0.92	0.94	6
Phe	1.09	1.02	1	0
Asp	0.97	0.94	0.88	12
Trp	0.92	0.88	0.83	17
Glu	0.97	0.92	0.9	10
His	0.93	0.92	0.94	6
Pro	1.1	0.97	0.97	3

* The areas are normalized to the initial storage conditions of the working solution. Amino acids are presented in ascending order of retention time.

**Table 6:** Chromatographic mobility of esterified amino acids obtained using triple quadrupole MS. Amino acids are presented in ascending order of retention time.

AA	Parent Ion, *m*/*z*	Retention Time, Min	Chromatographic Peak Height	Peak Width at Half Maximum, Min	Chromatographic Peak Area
Orn	189.16	1.003	2,328,761	0.060	9,761,179
Ala	146.10	1.089	2,134,894	0.067	8,632,169
Lys	203.17	1.453	1,660,029	0.057	6,460,292
Arg	231.18	1.742	2,262,781	0.061	9,666,924
Asn	189.12	2.026	103,235	0.058	408,153
Gly	132	2.078	105,593	0.061	427,161
Ser	162.11	2.181	925,478	0.059	3,700,371
Gln	203.14	2.323	369,800	0.065	1,619,872
Cit	232.16	2.767	1,250,933	0.059	5,037,170
Thr	176.13	2.915	512,854	0.060	2,088,165
Val	174.15	5.251	1,864,356	0.061	7,702,974
Tyr	238.14	5.321	2,051,976	0.062	8,525,253
Met	206.12	5.480	546,499	0.065	2,375,335
Leu	188.16	6.575	2,486,617	0.064	10,641,134
Phe	222.14	7.002	9,538,221	0.074	46,234,568
Asp	246.17	7.768	1,722,700	0.064	7,551,718
Trp	261.16	8.285	1,146,979	0.070	5,360,110
Glu	260.18	8.287	8,682,020	0.072	41,459,023
Bet	393.21	9.127	107,109	0.066	464,904

**Table 7:** Characteristics of the calibration curves of the analyzed amino acids obtained using triple quadrupole MS. Calibration curves were built by equal correlation within seven concentration levels, in three technical repetitions for each of the levels for glutamic acid, five concentration levels were used, except for two upper limit levels. Amino acids are presented in ascending order of retention time.

AA	k Slope Tangent	b Vertical Offset	r^2^ Correlation Coefficient	w Weight Factor	p Number of Points	L Number of Concentration Levels
Orn	0.011669	0.002859	0.9975	-	21	7
Ala	0.087133	0.025649	0.9931	-	21	7
Lys	0.264227	0.054483	0.9987	1/x	21	7
Arg	0.301652	0.589995	0.9876	1/x	21	7
Asn	0.018444	0.002325	0.9994	-	21	7
Gly	0.036322	0.013624	0.9731	1/x^2^	21	7
Ser	0.212549	0.151202	0.9989	-	21	7
Gln	0.261127	0.135219	0.9985	-	21	7
Cit	0.024455	0.042102	0.9965	1/x	21	7
Thr	0.102644	0.059708	0.9943	1/x	21	7
Val	0.373563	0.277502	0.9830	1/x	21	7
Tyr	0.291899	0.260901	0.9883	1/x	21	7
Met	0.093068	0.073779	0.9918	1/x	21	7
Leu	0.457484	0.768680	0.9513	1/x	21	7
Phe	0.963174	6.473994	0.8625	1/x^2^	21	7
Asp	0.358717	0.312286	0.9796	1/x	21	7
Trp	0.000045	0.000440	0.9687	-	21	7
Glu	2.399192	0.921144	0.9734	1/x^2^	15	5

**Table 8:** Chromatographic characteristics of carboxylic acids detected as a result of triple quadrupole MS analysis. Carboxylic acids are presented in ascending order of retention time (RT).

Carboxylic Acid	Chromatographic Characteristics *				*m*/*z*
	RT, Min	FWHM, s	As	A, *cnts × s*	
Succinic acid	2.7	2.94	0.88	55,540	165.05
Lactic acid	4.8	4.08	1.11	14,045	225.05
Pyruvic acid	6.0	3.42	0.93	1,152,626	202.05
Oxaloacetic acid	6.3	3.72	0.94	31,212	285.1
Isocitric acid	7.1	3.60	1.12	32,330	421.15
Taurine	7.6	3.78	1.08	228,750	271.1
Citric acid	8.3	3.72	1.07	277,775	269.05
α-Ketomethylbutyric acid	9.1	2.88	0.97	1,226,6593	179.05
α-Ketomethythiolbutyric acid	9.4	2.58	1.09	473,609	193.28
Malic acid	9.5	2.58	1.01	278,520	167.05
Fumaric acid	10.1	3.42	1.11	126,475	165.05

* The value of the mass charge state (*m*/*z*) is given for a monoprotonated ion (all carboxylic acids). The peak width is given to half the maximum height of the chromatographic peak (FWHM). The area is equal to the product of the peak width (As) and the number of elementary unit charges per sec (A).

**Table 9:** Characteristics of the calibration curves for the analyzed carboxylic acids obtained using triple quadrupole MS. Carboxylic acids are presented in ascending order of retention time.

Carboxylic Acid	k Slope Tangent	b Vertical Offset	r^2^ Correlation Coefficient	w Weight Factor	p Number of Points	L Number of Concentration Levels
Succinic acid	302.98	1654.2	0.9915	-	18	6
Lactic acid	73.312	1298.8	0.9667	-	18	6
Pyruvic acid	693.44	5815.8	0.9964	-	21	7
Oxaloacetic acid	156.49	2739.6	0.9866	-	21	7
Isocitric acid	145.06	18160	0.9602	1/x	21	7
Taurine	124.72	33.636	0.9554	1/x	18	6
Citric acid	165.17	1424.2	0.9990	-	21	7
α-Ketobutyric acid	217.54	1266.1	0.9987	-	21	7
α-Ketomethylbutyric acid	640.31	16027	0.9781	-	21	7
α-Ketomethythiolbutyric acid	265.23	1639.4	0.9976	-	21	7
Malic acid	155.42	1391.5	0.9820	-	18	6
Fumaric acid	561.54	4880.0	0.9959	-	21	7

**Table 10:** Quantitative measurements of amino- and carboxylic acids obtained using time-of-flight quadrupole MS from samples of primary human lung fibroblast cell cultures in two technical replications. Unit of measurement, μM, or 10^–6^ M, N/D—non-detectable. Amino acids and carboxylic acids are presented in ascending order of retention time. (For complete data, see Supplementary Materials, Table S4).

Primary Human Lung Fibroblast Cell Cultures												
Metabolite	0 h		24 h		48 h		72 h		1 Week		2 Weeks	
	Mean ± SD	CV,%	Mean ± SD	CV,%	Mean ± SD	CV,%	Mean ± SD	CV,%	Mean ± SD	CV,%	Mean ± SD	CV,%
Amino acids												
Ornithine	1.19 ± 0.00	0.24	10.49 ± 0.37	3.55	3.74 ± 0.02	0.59	1.29 ± 0.02	1.27	2.04 ± 0.08	3.84	7.44 ± 0.27	3.58
Alanine	2.10 ± 0.03	1.45	2.79 ± 0.10	3.45	1.25 ± 0.00	0.17	3.69 ± 0.04	1.02	1.36 ± 0.02	1.45	1.25 ± 0.02	1.64
Lysine	12.24 ± 0.52	4.27	15.18 ± 0.09	0.58	14.11 ± 0.00	0.03	17.47 ± 1.48	8.44	13.72 ± 0.80	5.83	6.25 ± 0.05	0.80
Arginine	19.25 ± 0.20	1.05	23.52 ± 0.05	0.20	20.34 ± 0.06	0.29	16.84 ± 0.06	0.38	17.07 ± 0.43	2.54	20.97 ± 0.49	2.35
Asparagine	18.67 ± 0.23	1.22	23.75 ± 2.28	9.59	20.21 ± 0.69	3.43	16.76 ± 0.47	2.79	16.83 ± 1.27	7.52	19.06 ± 0.25	1.31
Glycine	11.13 ± 0.03	0.24	12.88 ± 0.68	5.27	5.45 ± 0.13	2.39	7.65 ± 0.28	3.64	5.13 ± 0.09	1.68	1.13 ± 0.07	5.90
Serine	9.64 ± 0.75	7.81	10.62 ± 0.49	4.66	9.16 ± 0.17	1.84	6.72 ± 0.25	3.76	5.97 ± 0.02	0.36	5.25 ± 0.39	7.47
Glutamine	54.14 ± 2.31	4.27	50.12 ± 2.23	4.45	57.38 ± 4.00	6.98	44.28 ± 1.89	4.28	49.33 ± 3.70	7.49	49.34 ± 3.95	8.01
Citrulline	0.03 ± 0.00	6.53	0.06 ± 0.01	8.84	0.05 ± 0.00	6.87	0.02 ± 0.00	3.01	0.05 ± 0.00	8.16	0.10 ± 0.01	8.49
Threonine	11.58 ± 1.08	9.32	13.51 ± 0.90	6.63	13.86 ± 0.56	4.05	13.28 ± 0.15	1.15	14.27 ± 0.33	2.34	14.87 ± 0.08	0.56
Valine	1.45 ± 0.03	2.15	1.72 ± 0.04	2.22	1.71 ± 0.02	1.20	1.59 ± 0.03	2.18	1.56 ± 0.03	2.17	1.47 ± 0.06	3.84
Tyrosine	3.24 ± 0.09	2.82	3.77 ± 0.20	5.37	4.11 ± 0.08	1.98	4.71 ± 0.04	0.80	4.64 ± 0.01	0.15	4.41 ± 0.27	6.04
Methionine (sulfoxide)	1.39 ± 0.03	2.04	1.22 ± 0.03	2.27	0.91 ± 0.02	2.49	1.00 ± 0.01	0.78	5.93 ± 0.34	5.70	3.84 ± 0.03	0.88
Selenomethionine	0.71 ± 0.02	2.18	2.49 ± 0.06	2.50	3.03 ± 0.14	4.48	5.62 ± 0.10	1.77	6.37 ± 0.00	0.02	3.99 ± 0.13	3.14
Leucine	10.02 ± 0.34	3.37	10.31 ± 0.65	6.33	10.28 ± 0.18	1.78	10.10 ± 0.02	0.24	8.99 ± 0.60	6.72	8.26 ± 0.40	4.88
Isoleucine	10.21 ± 0.08	0.75	11.20 ± 0.61	5.44	10.86 ± 0.64	5.86	10.09 ± 0.00	0.02	9.80 ± 0.53	5.46	8.74 ± 0.28	3.15
Phenylalanine	2.13 ± 0.19	8.99	2.48 ± 0.09	3.62	2.44 ± 0.05	2.06	2.70 ± 0.14	5.15	2.71 ± 0.15	5.70	2.60 ± 0.10	3.84
Aspartate	7.79 ± 0.65	8.32	8.00 ± 0.25	3.17	5.83 ± 0.14	2.41	5.45 ± 0.36	6.60	4.49 ± 0.36	8.04	5.39 ± 0.16	3.03
Tryptophane	0.60 ± 0.00	0.82	0.59 ± 0.01	1.81	0.73 ± 0.02	2.15	0.62 ± 0.03	4.46	0.73 ± 0.06	8.46	0.75 ± 0.04	4.92
Glutamate	5.46 ± 0.24	4.39	11.50 ± 0.20	1.76	9.63 ± 0.19	2.00	8.93 ± 0.05	0.52	8.72 ± 0.45	5.19	8.73 ± 0.19	2.15
Betaine	1.46 ± 0.04	2.86	1.67 ± 0.10	5.96	1.66 ± 0.05	3.19	1.49 ± 0.11	7.22	1.51 ± 0.04	2.82	1.37 ± 0.08	6.02
Cysteine (sulfonate)	4.91 ± 0.28	5.63	3.86 ± 0.19	5.00	5.01 ± 0.11	2.18	3.57 ± 0.03	0.89	3.12 ± 0.15	4.72	3.27 ± 0.26	8.02
Glutathione (oxidized)	0.07 ± 0.00	1.08	0.59 ± 0.03	5.37	0.66 ± 0.01	1.83	0.98 ± 0.03	3.52	0.22 ± 0.00	0.98	0.65 ± 0.02	2.51
Proline	5.65 ± 0.01	0.16	6.79 ± 0.31	4.50	5.58 ± 0.30	5.40	8.75 ± 0.61	6.94	6.30 ± 0.09	1.41	7.97 ± 0.14	1.70
Histidine (2-oxo-)	1.93 ± 0.07	3.88	2.61 ± 0.16	5.98	2.38 ± 0.07	2.96	2.54 ± 0.10	3.80	2.00 ± 0.04	2.18	1.47 ± 0.07	4.43
Carboxylic acids												
Succinic acid	0.46 ± 0.01	2.62	0.79 ± 0.01	1.08	0.18 ± 0.00	2.82	0.14 ± 0.00	3.14	1.62 ± 0.03	1.62	0.25 ± 0.00	2.00
Lactic acid	20.02 ± 0.37	1.87	7.92 ± 0.21	2.68	5.83 ± 0.06	1.08	6.47 ± 0.08	1.27	18.45 ± 0.59	3.22	8.43 ± 0.28	3.36
Pyruvic acid	1.09 ± 0.02	1.43	0.16 ± 0.01	5.24	0.18 ± 0.00	1.62	0.05 ± 0.00	1.40	2.13 ± 0.01	0.37	0.17 ± 0.00	0.42
Oxaloacetic acid	1.84 ± 0.01	0.58	1.68 ± 0.03	1.81	0.41 ± 0.00	0.00	0.07 ± 0.00	3.14	3.07 ± 0.06	2.00	0.20 ± 0.00	0.00
Isocitric acid	0.003 ± 0.00	0.00	0.01 ± 0.00	0.00	0.001 ± 0.00	0.00	0.001 ± 0.00	0.00	0.11 ± 0.00	1.25	0.001 ± 0.00	0.00
Taurine	0.02 ± 0.00	2.89	0.03 ± 0.00	0.00	0.09 ± 0.00	4.88	0.07 ± 0.00	0.00	0.07 ± 0.00	2.08	0.12 ± 0.00	3.03
Citric acid	0.02 ± 0.00	0.00	N/D	N/D	N/D	N/D	0.001 ± 0.00	0.00	0.39 ± 0.00	0.73	N/D	N/D
α-Ketobutyric acid	0.45 ± 0.00	0.63	0.31 ± 0.00	0.23	0.33 ± 0.01	1.93	0.36 ± 0.01	1.95	0.75 ± 0.01	1.33	0.57 ± 0.00	0.37
α-Ketomethylbutyric acid	0.34 ± 0.02	4.56	0.21 ± 0.01	6.06	0.15 ± 0.00	2.29	0.04 ± 0.00	1.84	0.71 ± 0.01	1.70	0.05 ± 0.00	4.29
α-Ketomethylselenobutyric acid	N/D	N/D	0.06 ± 0.00	3.45	0.18 ± 0.01	4.43	0.28 ± 0.01	3.30	0.07 ± 0.00	3.87	0.19 ± 0.01	4.19
α-Ketoglutaramic acid	0.18 ± 0.00	2.77	0.03 ± 0.00	2.11	0.42 ± 0.02	4.71	2.53 ± 0.15	5.92	0.01 ± 0.00	0.00	1.74 ± 0.04	2.12
α-Ketoglutaric acid	0.001 ± 0.00	0.00	0.00 ± 0.00	0.00	0.03 ± 0.00	4.88	0.002 ± 0.00	0.00	0.001 ± 0.00	0.00	0.001 ± 0.00	0.00
Malic acid	0.21 ± 0.01	3.02	0.40 ± 0.00	0.88	0.02 ± 0.00	3.63	0.01 ± 0.00	0.00	2.08 ± 0.08	3.94	0.01 ± 0.00	0.00
Fumaric acid	1.93 ± 0.02	1.10	0.58 ± 0.01	1.33	0.13 ± 0.00	0.00	0.10 ± 0.00	3.63	1.50 ± 0.00	0.28	0.53 ± 0.01	1.74
cis-Aconitic acid	0.004 ± 0.00	0.00	0.03 ± 0.00	2.32	0.03 ± 0.00	0.00	0.08 ± 0.00	0.94	0.02 ± 0.00	0.00	0.07 ± 0.00	1.99
CoA	4.11 ± 0.03	0.69	4.55 ± 0.01	0.22	4.83 ± 0.02	0.48	3.54 ± 0.15	4.24	4.46 ± 0.05	1.17	4.59 ± 0.02	0.35
acetyl-CoA	0.04 ± 0.00	1.75	0.78 ± 0.01	1.91	0.90 ± 0.00	0.32	1.06 ± 0.01	0.80	0.83 ± 0.03	3.25	1.68 ± 0.07	4.09
Average CV, %	0.45 ± 0.00	0.63	0.31 ± 0.00	0.23	0.33 ± 0.01	1.93	0.36 ± 0.01	1.95	0.75 ± 0.01	1.33	0.57 ± 0.00	0.37
CV, ≥10%		0		0		0		0		0		0
CV, 5–10%		15		29		10		14		26		17
CV, 0–5%		85		71		90		86		74		83

**Table 11:** The main characteristics of the detection of signals of amino and carboxylic acids by the developed chromato-mass-spectrometric methods, based on the use of a mass analyzer with a triple quadrupole.

Parameters		Amino Acids	Carboxylic Acids
Peak asymmetry		1.096 ± 0.277	1.028 ± 0.065
Peak width at half height, s		5.601 ± 1.526	3.338 ± 0.513
Retention time deviation, %	L01 * 0.08 µM	99.28 ± 3.19	108.81 ± 2.50
	L04 0.63 µM	103.22 ± 4.07	101.42 ± 3.12
	L07 5.00 µM	97.58 ± 2.65	98.16 ± 2.19
Minimum SNR for L01		4437	279.84
Detection limit **		<4.8 nM	<0.5 μM
Estimated detection limit **		0.17 nM	7 nM
Analysis time, minutes		13.5	12

* L01, L04, L07—calibration levels total seven points; ** calculations made for alanine and lactate.

## Data Availability

The data presented in this study are available in article and Supplementary Material.

## Supplementary Materials

The following supporting information can be downloaded at: https://www.mdpi.com/article/10.3390/metabo13010016/s1, Table S1: List of standards. Table S2. Equimolar solutions of amino acids *. Table S3. Equimolar solution (1 mmol/L) of carboxylic acids. Table S4. Quantitative HPLC-MS/MS measurements of amino- and carboxylic acids in duplicates obtained from samples of primary human lung fibroblasts (HLF) cell culture. Unit of measurement, μM, or 10^–6^ M. N/D—non-detectable.
