# Supplementary material for: Chromatomass-Spectrometric Method for the Quantitative Determination of Amino- and Carboxylic Acids in Biological Samples
Source: Metabolites. 2022 Dec 22;13(1):16. doi: 10.3390/metabo13010016 (PMC9863782; doi:10.3390/metabo13010016)
Supplement: Supplementary file 1 [file metabolites-13-00016-s001.zip › metabolites-2065292-supplementary Revised/Kaisheva et al_Supplement_Revised.pdf]

Supplementary materials

Table S1. List of standards. (presented in ascending order of retention time, RT).

Name	Retention time, min	Empirical formula	Mw, Da
Amino acids			
Orn	1,003	$C_5H_{12}N_2O_2$	132.16
Ala	1,091	$C_3H_7NO_2$	89.09
Lys	1,548	$C_6H_{14}N_2O_2$	146.19
Arg	1,846	$C_6H_{14}N_4O_2$	174.20
Gly	2,128	$C_2H_5NO_2$	75.07
Asn	2,138	$C_4H_8N_2O_3$	132.12
Ser	2,299	$C_3H_7NO_3$	105.09
Gln	2,468	$C_5H_{10}N_2O_3$	146.14
Cit	2,747	$C_6H_{13}N_3O_3$	175.20
Thr	3,027	$C_4H_9NO_3$	119.12
Val	5,416	$C_5H_{11}NO_2$	117.15
Tyr	5,521	$C_9H_{11}NO_3$	181.19
Met	5,637	$C_5H_{11}NO_2S$	149.21
Ile	6.499	$C_6H_{13}NO_2$	131.17
Leu	6,611	$C_6H_{13}NO_2$	131.17
Phe	6,996	$C_9H_{11}NO_2$	165.19
Asp	7,884	$C_4H_7NO_4$	133.10
Trp	8,278	$C_{11}H_{12}N_2O_2$	204.23
Glu	8,352	$C_5H_9NO_4$	147.13
Bet	9,272	$C_5H_{11}NO_2$	117,15
Carboxylic acids			
Succinic acid	2.7	$C_4H_6O_4$	116.07
Lactic acid	4.8	$C_3H_6O_3$	90.08
Pyruvic acid	6.0	$C_3H_3O_3$	87.05
Oxaloacetic acid	6.3	$C_4H_4O_5$	132.07
Isocitric acid	7.1	$C_6H_8O_7$	192.12
Taurine	7.6	$C_2H_7NO_3S$	125.15
Citric acid	8.3	$C_6H_8O_7$	192.12
α-Ketobutyric acid		$C_4H_6O_3$	102.09
α-Ketomethylbutyric acid	9.1	$C_5H_9O_3$	117.05
α-Ketomethythiolbutyric acid	9.4	$C_5H_9O_3S$	149.02
Malic acid	9.5	$C_4H_6O_5$	134.09
Fumaric acid	10.1	$C_4H_4O_4$	116.07

Table S2. Equimolar solutions of amino acids* (presented in ascending order of retention time)

AA	Mw	Mass, mg	Volume, ml	Concentration, mg/ml	Concentration, mmol/L	Final concentration, μmol/L
Orn	132	1.3		2.6	9.85	984.85
Ala	89	3.1		6.2	35.06	3505.62
Lys	146	7.4		14.8	50.68	5068.49
Arg	174	5.1		10.2	29.31	2931.03
Asn	132	8.2		16.4	62.12	6212.12
Gly	75	4.4		8.8	58.67	5866.67
Ser	105	4.7		9.4	44.76	4476.19
Gln	146	3.2		6.4	21.92	2191.78
Cit	175	2.4		4.8	13.71	1371.43
Thr	119	1.7	0.5	3.4	14.29	1428.57
Val	117	3.7		7.4	31.62	3162.39
Tyr	181	1.9		3.8	10.50	1049.72
Met	149	3.0		6	20.13	2013.42
Leu/Ile	131	4.7		9.4	35.88	3587.79
Phe	165	4.3		8.6	26.06	2606.06
Asp	133	4.3		8.6	32.33	3233.08
Trp	204	2.1		4.2	10.29	1029.41
Glu	147	7.7		15.4	52.38	5238.10
Cys	121	4.4		8.8	36.36	3636.36

* Masses of arbitrary weights, calculated concentrations (mg/ml) and volumes of amino acid solutions for preparing an equimolar mixture.

Table S3. Equimolar solution (1 mmol/L) of carboxylic acids* (presented in ascending order of retention time)

Chemical name	Molecular formula	Mw	Sample weight, mg	Concentration, mmol/L
Succinic acid	$C_4H_6O_4$	116.07	7.60	130.96
Lactic acid	$C_3H_6O_3$	90.08	-	-
Pyruvic acid	$C_3H_3O_3$	87.05	1.60	36.76
Oxaloacetic acid	$C_4H_4O_5$	132.07	1.40	21.20
Isocitric acid	$C_6H_8O_7$	192.12	1.60	16.66
Taurine	$C_2H_7NO_3S$	125.15	12.50	199.76
Citric acid	$C_6H_8O_7$	192.12	5.40	56.21
α-ketobutyric acid	$C_4H_6O_3$	102.09	1.70	33.30
α-ketomethylbutyric acid	$C_5H_9O_3$	117.05	1.60	27.34
α-ketomethythiolbutyric acid	$C_5H_9O_3S$	149.02	1.00	13.42
Malic acid	$C_4H_6O_5$	134.09	3.80	56.68
Fumaric acid	$C_4H_4O_4$	116.07	0.90	15.51

* Arbitrary weights of carboxylic acids were dissolved in a fixed volume (0.5 ml) and the molar concentration of the resulting solution was calculated.

Table S4. Quantitative HPLC-MS/MS measurements of amino- and carboxylic acids in duplicates obtained using time-of-flight quadrupole MS from samples of primary human lung fibroblasts (HLF) cell culture.

Unit of measurement, μM, or 10^{-6} M. N/D – non-detectable. (Metabolites are listed in ascending order of retention time)

Points of cells growth: 0 point – HLF 01, 24 h – HLF 02, 48 h – HLF 03, 72 h – HLF 04, 1 week – HLF 05, 2 weeks – HLF 06.

Metabolite	HLF 01		HLF 02		HLF 03		HLF 04		HLF 05		HLF 06	
Amino acids												
Ornithine	1,185	1,189	10,755	10,228	3,726	3,757	1,274	1,297	1,989	2,100	7,627	7,250
Alanine	2,075	2,118	2,718	2,854	1,250	1,253	3,716	3,663	1,350	1,378	1,233	1,262
Lysine	12,614	11,874	15,243	15,118	14,117	14,111	16,430	18,516	14,289	13,157	6,211	6,282
Arginine	19,109	19,394	23,483	23,551	20,301	20,384	16,794	16,885	17,377	16,763	20,617	21,314
Asparagine	18,511	18,833	22,137	25,358	19,716	20,695	17,089	16,427	15,934	17,723	18,883	19,236
Glycine	11,109	11,147	12,398	13,358	5,355	5,539	7,842	7,448	5,195	5,073	1,079	1,173
Serine	9,107	10,171	10,266	10,966	9,037	9,275	6,545	6,903	5,987	5,957	4,976	5,531
Glutamine	52,509	55,775	48,541	51,698	54,549	60,211	42,936	45,615	46,713	51,941	46,549	52,136
Citrulline	0,031	0,034	0,060	0,068	0,049	0,054	0,024	0,023	0,049	0,055	0,094	0,106
Threonine	12,344	10,817	14,148	12,880	14,255	13,462	13,383	13,168	14,030	14,502	14,924	14,806
Valine	1,427	1,471	1,744	1,690	1,700	1,729	1,565	1,614	1,537	1,585	1,433	1,513
Tyrosine	3,301	3,172	3,912	3,626	4,050	4,165	4,682	4,735	4,638	4,648	4,224	4,601
Methionine (sulfoxide)	1,365	1,405	1,196	1,235	0,891	0,923	1,002	0,991	5,690	6,168	3,868	3,820
Selenomethionine	0,701	0,723	2,447	2,535	2,936	3,128	5,690	5,549	6,369	6,371	3,899	4,076
Leucine	10,260	9,782	10,770	9,847	10,405	10,146	10,084	10,118	9,417	8,563	8,548	7,978
Isoleucine	10,260	10,152	10,770	11,632	10,405	11,305	10,084	10,087	9,417	10,173	8,548	8,938
Phenylalanine	2,268	1,997	2,543	2,416	2,473	2,402	2,801	2,604	2,814	2,596	2,667	2,526
Aspartate	7,332	8,249	8,177	7,819	5,734	5,933	5,196	5,705	4,233	4,743	5,276	5,507
Tryptophane	0,605	0,598	0,594	0,579	0,736	0,714	0,638	0,599	0,771	0,684	0,773	0,721
Glutamate	5,628	5,289	11,643	11,356	9,761	9,489	8,958	8,892	9,040	8,400	8,601	8,867
Betaine	1,427	1,486	1,744	1,603	1,700	1,625	1,565	1,413	1,537	1,477	1,433	1,316
Cysteine (sulfonate)	5,110	4,719	3,997	3,724	5,083	4,929	3,590	3,545	3,219	3,011	3,457	3,086
Glutathione (oxidized)	0,066	0,065	0,615	0,570	0,648	0,665	1,008	0,959	0,219	0,216	0,636	0,659
Proline	5,653	5,640	7,005	6,573	5,793	5,367	8,318	9,177	6,365	6,239	7,871	8,062
Histidine (2-oxo-)	1,984	1,878	2,498	2,719	2,333	2,432	2,476	2,613	2,030	1,968	1,516	1,424

Carboxylic acids												
Succinate	0,450	0,467	0,793	0,781	0,172	0,179	0,138	0,132	1,598	1,635	0,244	0,251
Lactate	19,758	20,288	7,772	8,072	5,784	5,873	6,408	6,524	18,033	18,873	8,228	8,628
Pyruvate	1,075	1,097	0,168	0,156	0,177	0,173	0,050	0,051	2,123	2,134	0,168	0,169
Oxaloacetate	1,846	1,831	1,702	1,659	0,414	0,414	0,069	0,066	3,115	3,028	0,197	0,197
Isocitrate	0,003	0,003	0,010	0,010	0,001	0,001	0,001	0,001	0,112	0,114	0,001	0,001
Taurine	0,025	0,024	0,029	0,029	0,084	0,090	0,071	0,071	0,067	0,069	0,119	0,114
Citrate	0,016	0,016	N/D	N/D	N/D	N/D	0,001	0,001	0,388	0,392	N/D	N/D
α-Ketobutyrate	0,447	0,451	0,305	0,306	0,334	0,325	0,367	0,357	0,753	0,739	0,570	0,567
α-Ketomethylbutyrate	0,330	0,352	0,201	0,219	0,152	0,157	0,039	0,038	0,699	0,716	0,048	0,051
α-Ketomethylselenobutyrate	N/D	N/D	0,060	0,063	0,170	0,181	0,272	0,285	0,071	0,075	0,180	0,191
α-Ketoglutaramate	0,175	0,182	0,034	0,033	0,434	0,406	2,426	2,638	0,011	0,011	1,764	1,712
α-Ketoglutarate	0,001	0,001	0,001	0,001	0,030	0,028	0,002	0,002	0,001	0,001	0,001	0,001
Malate	0,206	0,215	0,399	0,404	0,019	0,020	0,014	0,014	2,024	2,140	0,010	0,010
Fumarate	1,916	1,946	0,590	0,579	0,129	0,129	0,095	0,100	1,505	1,499	0,523	0,536
cisAconitate	0,004	0,004	0,030	0,031	0,025	0,025	0,075	0,076	0,022	0,022	0,072	0,070
CoA	4,087	4,127	4,561	4,547	4,811	4,844	3,645	3,433	4,420	4,494	4,606	4,583
acetyl-CoA	0,040	0,041	0,765	0,786	0,895	0,899	1,061	1,049	0,808	0,846	1,630	1,727
